# The impact of polarity score on real option valuation for multistage projects

**DOI:** 10.1007/s11135-023-01635-6

**Published:** 2023-02-23

**Authors:** Antonio Di Bari, Domenico Santoro, Maria Antonia Tarrazon-Rodon, Giovanni Villani

**Affiliations:** 1 Department of Economics and Finance, University of Bari, Largo Abbazia S. Scolastica, 53, 70124 Bari, Italy; 2 Business Department, Universitat Autònoma de Barcelona, Campus de Bellaterra, 08193 Bellaterra, Cerdanyola del Vallès, Spain

**Keywords:** Broadband projects, Sentiment analysis, Information revelation, Real options

## Abstract

In most cases, the valuation of the investments characterized by various stages with a high level of uncertainty is done through the compound real option valuation (ROV). This decision making support can consider various types of uncertainty that can affects these investment phases, such as that linked to technology. Specifically, within the category of uncertain investments there are the broadband opportunities that can be valued as real options in order to quantify the risks associated with the investment. However, since ROV theory has no definitive way to determine model parameters based on market information, we propose one that can adjust them dynamically. In this paper, to include this aspect in the project valuation, we have unified the ROV with the sentiment analysis, a natural language processing technique that allows us to quantify the polarity of expressions in natural language numerically. In particular, the inherent risks related to the different phases of the project can be extracted from the information present in the surrounding environment and published in newspapers. From there, we obtain a sentiment score which, through appropriate manipulations, manages to modify the evaluation of the success probabilities of each stage. Then, we embed these success probabilities in the ROV in order to provide a valuation methodology that includes the impact of information on the investment decision.

## Introduction

There is no doubt that smart cities are, nowadays, the new frontier for public administration to improve citizens’ quality of life. Sometimes, the smart city concept can be embedded in a wider framework related to the sustainable development goals (SDGs) of the United Nations (UN). The improvements made by smart city projects can happen in different ways (by improving resources use, reducing emissions, upgrading water supply, etc.). A smart city is a place where traditional networks and services are made more efficient with the use of digital and telecommunication technologies for the benefit of its residents, and businesses.

The conception of a smart city may change according to the vision of researchers that apply this concept to lead some specific studies. For example, Caragliu et al. (2009) identified six dimensions of smart cities related to smart economy, smart mobility, smart environment, smart people, smart living, and smart governance. Another vision of smart cities highlights the concept of sustainability. Zygiaris (2013) found that a smart city could be related to “green”, “interconnected ”and/or “intelligent” concepts. Lam et Givens (2018) in their study defined smart cities as the “application of technology, data, and supporting tools and techniques to improve the quality of life”. The broadband economy can be viewed as a branch of smart city paradigms. Papacharissi and Zaks (2006) showed that developing a broadband society has become a very relevant goal for governments worldwide. This is because, nowadays, a rapid development in the telecommunications industry has happened, specifically in the diffusion of broadband internet access. Broadband technology represents the natural following step in internet evolution and diffusion by increasing speed and generating many innovations (Langdale 1997). These projects are characterized by risks and uncertainties in their decision-making process concerning upgrading and rolling their broadband networks. In one respect, these risks are referred to the characteristics of large infrastructure projects while, on the other hand, they are caused by unpredictable aspects such as potential competitor behavior, consumer demand, and rapid technological development (Fijnvandraat and Bouwman 2010). So, evaluating broadband technology investments, like other investment types in the Information and Communication Technology (ICT) fields, is challenging because it is characterized by high-level uncertainty and changing business conditions. For this reason, the classical Net Present Value (NPV) approach is insufficient to evaluate these types of investment since it cannot capture the complexity of broadband projects whose investment decisions need flexibility. Previous studies revisited the usefulness of the classical NPV since it cannot consider all the potential future projects related to the main investment that could increase its value (Ross 1995). Moreover, Alleman (2002) revised some more sophisticated tools of engineering economics, such as the Capital Asset Pricing Model (CAPM) or Decision Tree Analysis (DTA), explaining that neither of these methodologies can consider managerial flexibility in pricing. In this sense, Real Option Valuation (ROV) has spread as one of the most spanned methods in the literature and the industry to make a reliable assessment of complex, uncertain investments (Trigeorgis 1996). A practical introduction on how to adopt option pricing methods as useful decision support is provided by Smith and McCardle (1998). They evaluated oil and gas projects using option pricing methodologies in their work. Another interesting application is given by Secomandi and Seppi (2016) who applied real options in the energy field by considering risk-neutral and stochastic processes to capture the price dynamics.

Smart city renewable projects are comfortably evaluated with this approach. For example, Ha and Fujiwara (2015) used the real options approach as a theoretical framework to consider better the unpredictable aspects of technology and the market that affect firms’ investment and innovation decisions. In their work, they evaluated the R &D decision on a smart city project. Feng et al. (2016) applied ROV to evaluate the decisions to adopt a smart grid technology. Focusing on the ICT field, other examples of how ROV can be applied in broadband investment analysis are given by Elnegaard (2002) and Elnegaard and Stordahl (2002). In particular, their study referred to the technological upgrades from ADSL (Asymmetric Digital Subscriber Loop) to VDSL (Very High Data Rate Subscriber Loop). Angelou and Economides (2008) in their work analyzed the value of future investment opportunities deriving from initial infrastructure projects in the ICT area as real options by considering the competition risks. Tanguturi and Harmantzis (2006) used real options to value investment decisions affecting an Indian telecommunications service provider called Bharat Sanchar Nigam Limited (BSNL Ltd.). Again, Harmantzis and Tanguturi (2007) applied the real options methodology to defer the expansion from 2.5 G to 3 G network, and the expansion of a 2.5 G network by adopting Wi-fi technology. Charalampopoulos et al. (2011) studied the effect of regulatory scenarios on the timing of the investment decision to expand to a new network infrastructure investment by using the ROV. The interesting aspect of the ROV is that this methodology can be considered as a whole with other techniques. For example, Razgaitis (2003) applied the real options analysis and Monte Carlo Analysis to dealmaking processes in order to provide tools to price, negotiate and deal with technological and innovative R &D projects. On one hand, the real options analysis can set the model to price the possible results of a process, and, on the other hand, the Monte Carlo Analysis can determine the likely range of future possible results of this process. Other works have combined the ROV with game theory. For example, Smit and Trigeorgis (2007) analyzed the dynamic technology investments through a methodology that integrates real options with the game theory applied to strategic management and industrial organization. They consider the telecommunications and R &D projects by providing a tool to help potential investors to pursue strategic investment choices or options (such as when to invest, when to cooperate with other firms or when to compete. Thus, the studies summarized above explain the adequacy of the ROV in the valuation of uncertain projects such as broadband projects. Although the ROV allows to include the uncertainty in the project valuation through the pricing of managerial flexibility, it can only consider quantifiable financial parameters without explaining much about the related market information that can influence the investment decision. This is a limitation since, before pursuing a project, the investor should also consider the market information about that specific project by embedding the information revelation aspect in the project valuation. Posen et al. (2018) developed a methodology based on the behavioral theory of real options to face uncertain sequential decision-making problems. They combine the theory of real options with the feedback learning theory to support investment decisions by including firms’ beliefs regarding an investment adjusted for noisy feedback or contemporaneous uncertainty. Although this work represents the first attempt to include information in a project valuation, the substantial difference with the model proposed by us is in determining the uncertainty. The authors consider companies’ beliefs through values extracted from the market (through simulations). At the same time, our proposal is based on analyzing information from outside the company and expressed in natural language. To pursue this goal, we combine the ROV with sentiment analysis. This Natural Language Processing (NLP) task allows analyzing human opinions through sentences expressed in natural language (Nasukawa and Yi 2003), classified according to polarity, irony, and personality (personal and impersonal). For the specific application of increasing the amount of information, we only considered the analysis of natural language sentences according to their polarity in the strict sense (positive, neutral, or negative), excluding, for example, the analysis of the irony or personality that may not add any information. Thanks to the development of neural networks and the introduction of Transformers (Vaswani et al. 2017), Bidirectional Encoder Representations from Transformers (BERT) and its evolutions have become state-of-the-art among NLP models. In particular, this encoder architecture for training uses Masked Language Modeling (MLM), which consists in masking 15% of the tokens that make up the corpora and predicting them) to understand the language of a specific domain, and the Next Sentence Prediction (NSP, which consists in predicting whether a sentence is after the previous one or random). To evaluate the information of the domain in question (the telecommunications sector and broadband systems), we consider a particular version of BERT, called AlBERTino (Colasanto et al. 2022). This model, based on the AlBERTo model (Polignano et al. 2019), was trained on a corpus of Tweets (TWITA dataset, Basile et al. (2018)) collected between 2012 and 2018 in Italian and containing information belonging to an extensive range of domains (from politics to information technology, including that of our interest) and fine-tuned by adding a layer trained on financial information. This NLP Italian model allows us to acquire knowledge from newspaper articles relating to pre/post situations that may occur around a broadband project.

Generally, sentiment analysis is applied to financial options theory, as evidenced by Han (2008) analyzing the stock market, determines a pattern between the polarity and volatility changes, like Hao (2017) which demonstrates how through the use of sentiment analysis it is possible to reduce pricing errors, or by Wang et al. (2022) who have studied the relationship between sentiment and the price of put options. Some authors, such as Posen et al. (2018); Hartmann and Hassan (2006) have developed the theory of real options in combination with additional elements to increase the amount of information available. However, a link between ROV and sentiment analysis through NLP is not directly present in the literature since the increased available information is generally incorporated in the models’ classical parameters. In this paper, we extend the existing literature to combine the Real Option Valuation (ROV) with a particular Bidirectional Encoder Representations from Transformers (BERT, Devlin et al. (2019)) model that will allow us to capture the polarity from some newspaper articles, introducing the ROBERT (Real Option BERT) theory. This way, we can consider information such as possible results/problems related to a broadband system outside the investment. Thanks to this, we can adjust the evaluation of the different stages of the investment. This work is organized as follows: Sect. 2 provides the methodological framework; Sect. 3 proposes an ideal case study based on likely data; Sect. 4 presents the concluding remarks.

## Methodology

In this section, we provide a methodology suitable for the characteristics of broadband investments. We proceed, first, by explaining the sequential nature and risks of a broadband project in Sect. 2.1, by describing the importance of sentiment score and how it is possible to link it to the probabilities of success in Sect. 2.2, to move, next, to present the broadband project as a compound real options model in Sect. 2.3.

### Broadband characteristics: sequential nature and risks

**Fig. 1:**
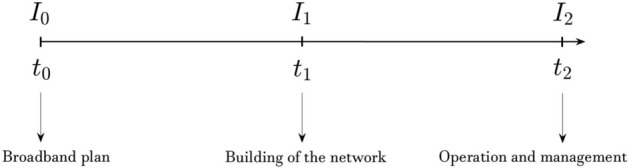
Stages of a typical broadband project

Broadband projects are characterized by sequential steps that start with a broadband plan. In the first step, the sponsor needs to think strategically about the project; therefore, there is a first overall assessment. We can include various activities such as mapping infrastructure needs, making a possible financial plan and a business model, developing deployment planning, and procurement preparation in this phase. The broadband plan is a relevant point to make an overview of the socioeconomic and demographic features of the territory. Moreover, it contains information regarding the definition of the project’s goals in terms of the demand for high-speed broadband by the population. However, the market’s real conditions during the project’s lifetime can change drastically from the prevision made in this first general assessment. After this step, if the conditions appear favorable, the manager should decide to undertake the first real investment phase related to the building of the network and seek funding from potential investors. Successively, the investment process proceeds with the operation and management stage.^1^ For each step, different amounts of cost are associated, and, in the operating phase, it is expected to recover all the costs thanks to the revenues generated by the broadband project. Specifically, the net revenues, in this case, are referred to cash inflows directly paid by users for the goods or services provided by the operation. For example, charges are borne directly by users for the use of infrastructure. The sequential stages are summarized in Fig. 1 where $I_{0}$, $I_{1}$ and $I_{2}$ represent the different amounts of investment required, respectively, for a broadband plan, building phase and operating phase. These investments are pursued respectively at time $t_0$, $t_1$, and $t_2$. Since $t_2$ represents the final period, it corresponds with the project’s maturity *T*.

Considering the sequential nature of these investments, it is easy to understand the need to embed the managerial flexibility insight in their valuation to allow investors to continue in the following stages only if the previous stages are profitable. This approach reduces the risk of huge losses and makes the project realistically attractive.

Regarding the broadband risks, we can divide them into operational, market, financial, and political risks (see Feasibility Report for a Community Network (2020)). The operational risks are related to the potential failure of tasks required for the investment. The market risks could be various. For example, the investor could face a new internet service provider entering the market. This situation can generate a fight of price between the incumbent and competitor to acquire a higher market share. In this sense, both could fix a low price to appear attractive to customers. In this way, although they should aim to acquire a high market share, the revenues will reduce. Moreover, the investor could face financial risks since the fiber networks are quite expensive and there is no guarantee that a new fiber business can generate cash inflows to be self-sufficient. Another example of financial risk is related to the “cost of success”. This happens when the internet service provider, facing a lack of financial resources, cannot acquire new customers, although they would buy his service, for the high acquisition cost. Political risks are related to the behavior of future politicians in broadband investments. This short overview of the risks highlights the importance of including volatility in the project valuation. The project value of the broadband project can change over time due to these risks, which we include in our valuation by using volatility parameters.

### Sentiment score and probability of success

What we do, at this point, is to link the value of the sentiment score to the probability of success present in each stage. So, for example, considering the $S_1$ stage of construction of the network, the probability of success that allows passing to stage $S_2$ of operation and management can be “adjusted" based on information extracted from newspapers. Suppose we want to start an investment in broadband infrastructure. In this case, the probability *p* of passing from stage $S_0$ to $S_1$ can be modified considering the evolution of the already existing broadband infrastructures (investments by governments, impossibility to build, and so on), such as those described in the newspaper articles. The articles considered in this case have a telecommunications thread (as highlighted by the keywords in Table 1), and the fact that their publication took place in critical Italian newspapers ensures high visibility. A report is added directly from Cisco, the manager of most of the infrastructures. We have considered the articles described in Table 1.

**Table 1:** Description of the newspaper articles used to calculate the polarity score

Article	Description	Structure	Keywords
Broadband and satellities$^2$	The main topic concerns the growth of the space economy by the European Union and the advantages that this can bring; expansion is also linked to the development of broadband infrastructures	This article, published on 1/29/2022 on *Corriere della Sera*, consists of 716 words and is divided into 28 periods	Telecommunications, Space + Aeronautics Agencies, Spacecraft
Cisco Bradband$^3$	The main topic concerns the analysis of broadband infrastructures present on the Italian territory and how these can affect agile work (remotely) and digital inclusion	This report, published on February 27, 2022, consists of 649 words and is divided into 19 periods	Work + Family, Telecommunications, Internet connection
Security and broadband$^4$	The main topic concerns the need to strengthen the Italian IT security perimeter (also following the events that occurred during the conflict in Ukraine), specifying how the legislator pushes toward a reorganization in the field of 5 G broadband electronic communication	This article, published on 4/7/2022 on *IlSole24Ore*, consists of 947 words and is divided into 20 periods	Security, Telecommunications, Digital attack
Governments and broadband$^5$	The main topic concerns the flop of one of the tenders for the enhancement of the broadband infrastructure, especially in the peripheral areas of the Italian peninsula, with funding from the European Union	This article, published on 5/15/2022 on *IlSole24Ore*, consists of 628 words and is divided into 18 periods	UE Commission, Telecommunications, 5 G

Francesca Basso, Pasquali: “Telespazio punta ai progetti europei Satelliti per banda larga e agricoltura”, January 29, 2022, corriere.it

Cisco, Broadband Index 2022, February 27, 2022, available at https://www.cisco.com/c/m/en_us/solutions/broadband/broadband-index.html#blade-1

Colajanni M., Finocchiaro G., Mele S., and Pollicino O., Sicurezza, settori strategici e banda larga: come cambia la protezione dei dati, April 7, 2022, ilsole24ore.com

Carmine Fotina, Banda larga, va deserta gara 5 G da 1 miliardo con fondi del Pnrr, May 15, 2022, ilsole24ore.com

The average sentiment (polarity) score, $\gamma \in [-1, 1]$, is determined by considering the sentences present in each article. Generally, a sentence goes from point to point, but since in journalistic language, there are often concise sentences whose polarity is neutral, we have aggregated the shorter sentences with the rest to obtain a certain number of sentences for each article, such as described in the previous table. In particular, a sentiment score was determined for each period, a first average represents the polarity of the article, and the average of these latter values represents the global score that will be used to “adjust" the probability of success. For example, for the report *Cisco Broadband*, the first sentence consists of the following sentence: *“La crescita sociale ed economica di un Paese è legata a doppio filo con un accesso universale, affidabile e veloce a internet."*, whose polarity is positive with $\gamma = 0.53$; while the second sentence period consisting of the sentence *“E’ questo il principale dato che emerge dall’edizione 2022 del Broadband Index di Cisco, uno studio condotto ogni anno su quasi 60.000 lavoratori di trenta Paesi (fra cui l’Italia) chiamati a rispondere sulla qualità della banda larga domestica."* has a neutral polarity with $\gamma =0.05$. Furthermore, since the sentiment score expresses whether a sentence is negative, neutral, or positive, we can break $\gamma$ so that, if $\gamma \in [-1, 0)$, then the sentence represents negativity; if $\gamma \in [0, 1]$, then represents positivity; while for values close to 0, the score represents neutrality. Considering the articles described above, we obtain the sentiment and global scores shown in Table 2.

**Table 2:** Sentiment score for each article and global (average) score

Article	Polarity	$\gamma$
Broadband and satellities	Positive	0.14
Cisco Bradband	Positive	0.36
Security and broadband	Positive	0.15
Governments and broadband	Positive	0.14
Global		0.20

The choice of articles was made randomly, and to avoid news polarization, different journals were considered. However, since there is not much specific news on the broadband sector, we have limited ourselves to considering articles that allow us to extrapolate helpful information on the future of a broadband system. Observing how all the articles have a positive score, we infer that the broadband infrastructure sector is undergoing strong development and is encouraged by institutions and companies.

At this point, to understand how to use the sentiment score to adjust the probability of passing from one stage to another, we must refer to the theory introduced by Dias (2005), who modeled how uncertainty impacts on the technical success of a particular project. In his work, which is part of the Value of Information (VOI) theory, Dias considers the technical uncertainty associated with a project and defines a measure (so-called *learning measure*) that takes into account the possible information revealed (e.g., in the real options settings like a learning options exercises), and how this can eliminate part of the uncertainty, modifying the probability of success. Returning to the case of a single firm characterized by a multistage project (each of which with probability $p_i$ (Cassimon et al. 2004)), it is possible to define two Bernoulli variables $S_1$ and $S_2$ with the following probabilities:

$$\begin{aligned} S_1 = {\left\{ \begin{array}{ll} 1 &{} p\\ 0 &{} 1 - p \end{array}\right. }, \quad S_2 = {\left\{ \begin{array}{ll} 1 &{} q\\ 0 &{} 1 - q \end{array}\right. } \end{aligned}$$

where $S_1$ is the company’s performance in a given stage (the so-called *signal* in Dias framework) and $S_2$ is the performance in the next stage, correlated to the previous one. Thus, by considering the phases described in Sect. 2.1 we can identify as $S_1$ the stage of building the network and as $S_2$ the stage of operation and management.

Investing in information (information revelation process) to reduce uncertainty corresponds, in this case, to study the relationship between the two previous variables and, therefore, the correlation of a bivariate Bernoulli variable. As defined by Dias (2005), revelation distribution is the conditional expectation of information to the exercise of a learning option. In this way, it is possible to define the random variable associated with the revelation distribution with $R_{S_2}(S_1) = E[S_2 \mid S_1]$, whose probability density $p(R_{S_2})$ has some properties related to the mean $E[R_{S_2}] = E[S_2]$ and variance $Var[R_{S_2}] = Var[S_2] - E[Var[S_2 \mid S_1]]$. Considering two Bernoulli variables, the expected percentage of variance reduction is

1 $$\begin{aligned} \eta ^2(S_2 \mid S_1) = \frac{Var[R_{S_2}]}{Var[S_2]}, \end{aligned}$$

a particular learning measure that (Dias 2005) always exists, is generally asymmetric and normalized to the unit interval, is invariant under linear transformations, and if $S_1$ and $S_2$ are independent, then $\eta ^2(S_2 \mid S_1) = \eta ^2(S_1 \mid S_2) = 0$.

To study the effect of performance in one stage on the next, we consider the joint distribution between $S_1$ and $S_2$ (bivariate Bernoulli distribution) characterized by the probabilities *p*, *q*, and the joint probability of success $\eta ^2$. The revelation distribution is characterized by two scenarios, which Dias (2005) indicates with $CF^+$ and $CF^-$, but which, in this case (to maintain a notation conforming to the rest that does not create confusion), we indicate in the following way:

2 $$\begin{aligned} q^{pos}&=Prob[S_2=1 \mid S_1=1] = E[S_2 \mid S_1 = 1], \end{aligned}$$

3 $$\begin{aligned} q^{neg}&=Prob[S_2=1 \mid S_1=0] = E[S_2 \mid S_1 = 0]. \end{aligned}$$

Thus, we have introduced two Bernoulli variables linked by the $\eta ^2$ parameter that respond to the conditions of Theorem 4 (Dias 2005), for which the Fréchet-Hoeffding bounds are in terms of $\eta ^2$, the measure is symmetric, $\eta ^2=0$ iff $S_1$ and $S_2$ are independent, the learning measure is equal to the correlation coefficient $\eta ^2(S_2 \mid S_1) = \rho ^2(S_2, S_1)$, and the revealed success probabilities in the case of positive dependence are:

4 $$\begin{aligned} q^{pos}&=Prob[S_2=1 \mid S_1=1] = q+\sqrt{\frac{1-p}{p}}\cdot \sqrt{q(1-q)} \cdot \rho (S_2,S_1), \end{aligned}$$

5 $$\begin{aligned} q^{neg}&=Prob[S_2=1 \mid S_1=0] = q-\sqrt{\frac{p}{1-p}}\cdot \sqrt{q(1-q)} \cdot \rho (S_2,S_1) \end{aligned}$$

(for negative dependence inverting the sign after *q*). Considering $S_1$ and $S_2$ *exchangeable* Bernoulli variables (as often happens in reality), it is possible to simplify the Fréchet-Hoeffding bounds to $0 \le \eta ^2 \le 1$, and the revealed success probabilities to

6 $$\begin{aligned} q^{pos}&=q + (1-q)\eta , \end{aligned}$$

7 $$\begin{aligned} q^{neg}&=q - q\eta . \end{aligned}$$

The $\eta ^2$ parameter represents, as mentioned, the revealed information generated upon an event’s occurrence and allows to modify the probability of success (performance) of a stage. Therefore, by analyzing the news relating to the project of interest (broadband in our case), we can associate the information revealed with the $\gamma$ sentiment score determined through AlBERTino. As introduced in Santoro and Villani (2022), we can set $\eta ^2=\gamma$ and verify how the conditions above characterizing a measure are almost all met, except for Fréchet-Hoeffdind bounds. To solve this problem, we can perform some manipulations so that $0 \le \gamma \le 1$ for both positive and negative scores. This new parameter, called $\gamma _{adj}$, can be obtained using the sigmoid function (logistic function) computed as

8 $$\begin{aligned} \gamma _{adj} = \frac{1}{1+\exp ^{-\gamma }} \in (0,1), \end{aligned}$$

which, thanks to its performance, allows to maintain an idea of the positivity/negativity of the score. With this parameter, the revealed success probabilities become:

9 $$\begin{aligned} q^{pos}&=q + (1-q)\sqrt{\gamma _{adj}}, \end{aligned}$$

10 $$\begin{aligned} q^{neg}&=q - q\sqrt{\gamma _{adj}}, \end{aligned}$$

for positive dependence; in the negative case, inverting the signs after *q*.

### The mitigation of broadband riskiness by embedding sentiment score analysis in real options approach

The valuation of smart city projects, specifically broadband projects, can be viewed as a series of sequential investments related to different stages in their lifetime period. These stages are not independent of each other but, quite the opposite, the investment process is not able to proceed in the following stage if the previous stage presents a kind of failure. This logic can be captured by compound ROV. Previous studies used this approach to valuate sequential investments. For example, Cassimon et al. (2004) used compound ROV for valuing a new drug application since pharmaceutical investments can be viewed as a series of consecutive phases from R &D to commercialization. Again, Hauschild and Reimsbach (2015) in their study proposed a simplified way to valuate sequential R &D investments by using a binomial approach. In this section, we analyze the broadband project through compound option pricing in discrete time in order to estimate this investment affected by a high rate of uncertainty. This refers, particularly, to the volatility of this type of project due to the unstable business conditions that they undergo.

We apply the usual binomial lattice model that is adequate to manage the real-case applicability. The analysis starts by calculating the market value of the project, in other words, the gross present value (*PV*) that acts as the underlying asset of the compound option, as shown in Eq. 11.

11 $$\begin{aligned} PV=\sum _{n=0}^{n=T}\frac{Rev_{n}-OC_{n}}{(1+r)^n} \end{aligned}$$

As we see, the *PV* is equal to the net value given by the discounted difference between revenues (*Rev*) and operating costs (*OC*). Considering the volatility ($\sigma$) of broadband projects, we can state that they evolve as a binary random walk through two movements: up (*u*) and down (*d*) that are calculated as follows (Cox et al. 1979):

12 $$\begin{aligned}{} & {} u=e^{\sigma \sqrt{\Delta t}} \end{aligned}$$

13 $$\begin{aligned}{} & {} d=e^{-\sigma \sqrt{\Delta t}} \end{aligned}$$

Consequently, following option theory, the PV evolution - that is the underlying asset of the real option under analysis - is calculated as:

14 $$\begin{aligned}{} & {} PV \cdot u \end{aligned}$$

15 $$\begin{aligned}{} & {} PV \cdot d \end{aligned}$$

Each movement of the underlying asset is associated with a risk-neutral probability: respectively, *q* for upward-going movement *PVu* and $(1-q)$ for downward-going movement *PVd*. The value of *q* is calculated considering the absence of arbitrage, i.e. under fair pricing conditions, as follows:

16 $$\begin{aligned} q=\frac{(1+r_{f})^{\Delta t}-d}{u-d} \end{aligned}$$

We implement the insight of simple ROV in which the investor should proceed with further next stages only if the evolution of PV is higher than $I_{s}$ in the current stage *s*. Otherwise, s/he would give up investing and the project value would become nil ($=0$). This allows us to price the managerial flexibility value and to embed the optionality in the broadband project valuation.

The analysis starts from the maturity *T* up to the initial time period ($t_0$) following the backward induction procedure along the binomial lattice model. This allows the investor to make an ex-ante investment decision about the future project performance. We move at the end of the operating phase, *T*, where the simple option value is calculated as follows:

17 $$\begin{aligned} s_{T}^{a(+);(b-a)(-)}(PV_T, I_2, 0)=\max [PV\cdot u^{a}d^{b-a}-I_2;0] \end{aligned}$$

where $a=0,...,b$ and $b-a$ represent, respectively, the up and down movements of the underlying assets along the binomial tree. This payoff implies that the investor should pursue the operating stage only if the project value evolution is higher than cost $I_2$ for operating activity. However, this payoff is incomplete since it does not take into account the information revelation that the investor can obtain by pursuing the previous stage, and so the building of the network. During the operation, the investor can learn about positive (*pos*) or negative (*neg*) information revelation regarding the project value evolution. This information can influence the following investment stages by increasing or decreasing the project value. The inclusion of information revelation into ROV has been made by Di Bari and Villani (2022) in a recent study. In their work, they considered the information revelation obtained by the first mover advantage in the competition case. In this case, we consider the information revelation that a previous investment stage gives to the following stage according to its successful or disastrous performance. Differently from the study of Di Bari and Villani (2022), in this work, the information revelation probabilities have been calculated considering the polarity of information coming from the outside, through Sentiment Analysis with AlBERTino. Thus, by including the information revelation probabilities in Eq. 22, we can rewrite it by using the notation $s_T^{pos}$, in the case of positive information revelation, $s_T^{neg}$, in the case of negative information revelation, and $\tilde{s}_T$ to identify the real option value that considers both scenarios. In fact, the value of $\tilde{s}_T$ is calculated as follows:

18 $$\begin{aligned} \tilde{s}_T=p\cdot s_T^{pos} +(1-p)\cdot s_T^{neg} \end{aligned}$$

where

19 $$\begin{aligned}{} & {} s_T^{pos}= \max [q^{pos}\cdot PV\cdot u^{a}d^{b-a}-I_2;0] \end{aligned}$$

20 $$\begin{aligned}{} & {} s_T^{neg}= \max [q^{neg}\cdot PV\cdot u^{a}d^{b-a}-I_2;0] \end{aligned}$$

The probabilities related to positive and negative information revelation, $q^{pos}$ and $q^{neg}$, have been calculated following Sect. 2.2.

Once calculating the option values at maturity *T*, the analysis proceeds backward until the final time period of the previous stage ($t_1$) which is the building of the network:

21 $$\begin{aligned}{} & {} \tilde{s}_{t_{1}}^{a_1(+);(b_1-a_1)(-)} ( PV_T,I_{2},T-t_1 )=\nonumber \\{} & {} \frac{\sum _{a_2=0}^{b_2} \left( {\begin{array}{c}b_2\\ a_2\end{array}}\right) \cdot \pi ^{a_2}\cdot (1-\pi )^{b_2-a_2} \cdot \tilde{s}_T^{(a_{2}+a_{1})(+), b-a_{2}-a_{1}(-)}( PV_T,I_{2},0)}{(1+r_f)^{T-t_1}} \end{aligned}$$

where $b_1=\frac{t_1}{\Delta t}$, $b_2=\frac{T-t_1}{\Delta t}$, $a_1=0,...,b_1$ and $a_2=0,\ldots ,b_2$. The parameter $\tilde{s}_{t_{1}}$ represents the value that the project assumes at time $t_1$. In this time period, the investor faces a new investment cost $I_1$ related to the construction of the network, and so s/he should consider a new payoff:

22 $$\begin{aligned} \tilde{c}_{t_1}^{a_1(+);(b_1-a_1)(-)}(\tilde{s}_{t_{1}}, I_1, t_{1}-t_{0})=\max [\tilde{s}_{t_{1}}-I_1;0] \end{aligned}$$

To know the project value at time $t_0$, we follow the same backward induction procedure by subtracting the new cost $I_1$:

23 $$\begin{aligned} \tilde{c}_{t_0} =\frac{\sum _{a_1=0}^{b_1} \left( {\begin{array}{c}b_1\\ a_1\end{array}}\right) \cdot \pi ^{a_1}\cdot (1-\pi )^{b_1-a_1} \cdot \tilde{c}_{t_1}^{a_1(+);(b_1-a_1)(-)}(\tilde{s}_{t_1},I_{1},T-t_1)}{(1+r_f)^{t_1-t_0}} \end{aligned}$$

The parameter $\tilde{c}_{t_0}$ represents the broadband project value at time $t_0$. The value of $\tilde{c}_{t_0}$ should be multiplied by the success probability of the building of network *p* and, by subtracting $I_0$ (the investment for the broadband planning), we can obtain the final Real Option (RO) value:

24 $$\begin{aligned} \hbox {RO value}=-I_0+p\cdot \tilde{c}_{t_0} \end{aligned}$$

## Case study

In this section, we present a standard case study referring to a broadband investment located in Europe and projected into the future considering a period between 2020 and 2030, i.e., we face a projection of 10 years. The choice of this period has been made considering that the analysis is forecasted into the future and we find it interesting to start in 2020, the year of the Covid-19 pandemic outbreak. With this tragic event that has had so many different consequences in our lives, people have gotten used to working from home using the so-called “smart working”and, thus, the importance of a high-speed internet connection has raised significantly worldwide. As already mentioned, our case study is based in Europe. In Sect. 3.1, the extrapolation procedure of revenues and costs values is highlighted, in the following Sect. 3.2 there is the calculation of the amount of investment in each phase, in Sect. 3.3 the polarity score is implemented in the ROA with the previous data, finally in Sect. 3.4 implications and limitations of this embedding are presented.

### Extrapolation of revenues and costs values

We extrapolate the values of revenues and operating costs from the *Feasibility Report for a Community Network*. *Farmington/Farmington Hills Michigan* by Finley Engineering CCG Consulting, dated November 2020. Since the data available in the report are data accumulated after a period of 20 years, we could not use them for our analysis referred to a period of 10 years. The revenues that we considered in the report are equal to 261.31 $ that we convert into Euros becoming 213.43 €. While the operating costs converted in Euros are equal to 78.38 € organized as shown in Table 3.

**Table 3:** Operating costs after 20 years

Item	Cost (million of €)
Cost of goods and service sold	7.09
Operating expenses	65.58
Interest expense	1.16
Income tax	4.55
Total	78.38

The ratio between operating costs and revenues after 20 years is equal to 0.3672. We have assumed to take back the available data to a period of 10 years rather than 20, by applying some percentages of the longer period. In this sense, the report showed different analyses that consider the comparison between financial results after 10 years, and then after 20 years. According to a general overall view of the report, we can state that the accumulated revenues after 10 years can approximately correspond to $30\%$ of the accumulated revenues after 20 years. On the basis of this analysis, we have applied a percentage of $26\%$ on the operating costs related to 20 years to obtain the same costs referred to a period of 10 years and a percentage of $27\%$ on the revenues. The choice of these percentages has been made assuming that the ratio between operating costs and revenues should be higher after 20 years considering the aspect of the “cost of success” explained in Sect. 2.1. The assumption stemming from the consultancy and engineering report that we made is that it is costly to add new customers if the project exceeds the expected results. The values related to a period of 10 years are summarized in Table 4.

As shown in Table 4, we use revenues value after 10 years equal to 57.63 million Euros and operating costs value equal to 20.38 million Euros. Thus, we obtain the ratio between operating costs and revenues after 10 years equal to 0.3536, slightly lower than 0.3672, which is the same ratio extended for a 20 years period. So, in this sense, we respect the condition related to the presence of the “cost of success”.

**Table 4:** Revenues and operating costs after 10 years and 20 years

Item	Values after 20 years	Values after 10 years
Revenues	213.43	$213.43\cdot 0.27= {\EUR } \ 57.63$ million
Operating costs	78.38	$78.38\cdot 0.26= {\EUR } \ 20.38$ million
Ratio	0.3672	0.3536

### Data collection

The data that we use, related to a consultancy and engineering Feasibility Report for a Community Network, is dated November 2020, during the pandemic period. We provide a simplification using likely data related to a broadband structure to implement a multi-stage ROV modeled as compound options. The data are chosen considering the Feasibility Report for a Community Network^2^ to which we apply some changes in order to convert US dollar in Euros. This project is developed by an ISP (Internet Service provider) that wants to provide the market by selling the Internet Fiber of Download Speed of 1 Gbps. We assume that the price of Internet 1 Gbps is equal to € 81.67 and that modems are leased at € 8.17 per month. The present value of this project (*PV*) is equal to € 11.94 million which acts as the underlying asset. This value has been calculated considering the difference between the revenues and operating costs values (the latter contains the cost of goods and services sold, operating expenses, interest expenses, and income taxes) on the basis of the Feasibility Report for a Community Network,^3^ after converting the items into Euros. Specifically, the Revenues after 10 years amount to € 57.63 million and the operating costs after the same period amount to € 20.38 million.^4^ Since the Revenues and Operating costs are accumulated values over 10 years, we have discounted them at time 0, i.e. 2020, at a cost of capital (*r*) of $12.05\%$. The choice of $r=12.05\%$ is a realistic rate under current circumstances. Besides, it is to be found also in previous analyses such as the one undertaken by Sinha and Gupta (2011). So, the discounted difference between revenues and operating costs is calculated as follows:

25 $$\begin{aligned} PV_{0}=\frac{Rev.}{(1+r)^{10}} - \frac{Op. costs}{(1+r)^{10}}= \frac{57.63}{(1.1205)^{10}} - \frac{20.38}{(1.1205)^{10}}=18.47 - 6.53= 11.94 \end{aligned}$$

At this point, we can identify the amount of investment for each stage. We start by considering the costs pursued at time $t_{1}=1$, the year of building the network ($I_{1}$). According to the engineering study, we assume that $I_{1}= {\EUR } \ 10.725$ million organized as shown in Table 5.^5^

**Table 5:** Costs of building the network

Item	Cost (million of €)
Fiber	6.36
Drops	1.33
Electronics	2.51
Huts	0.095
Operational assets	0.43
Total	10.725

We hypothesize that the initial investment pursued at time $t_0=0$ for the broadband plan $I_{0}$ is represented by a percentage of total costs, that we assume to be equal to $3\%$ of the total costs (construction costs + operating costs$=10.7250+6.5326=17.2576$). And so, we obtain $I_{0}={\EUR } \ 0.5177$ million. With the same logic, we calculate the amount of money required for the operation phase $I_{2}$ as a percentage of $2\%$ of the total costs, and so $I_{2}={\EUR } \ 0.3452$ million. Now, we can proceed to analyze the operational flexibility by including AlBERTino’s sentiment score in the broadband project valuation.

### Implementation of real option approach by embedding sentiment score and probability of success

Broadband projects are risky investments as we have shown in Sect. 1. In other words, they are characterized by a high level of volatility ($\sigma$). In our case study, we assume this volatility to be equal to $46.96 \%$ chosen by considering Tanguturi and Harmantzis (2006) and Di Bari and Villani (2022). To calculate $\sigma$, these studies used the historical price movements of the Bombay Stock Exchange Technology, Media and Telecom Index (BSE TECk).

Applying a value of $\sigma$, we can calculate the parameters of up and down considering yearly movements ($\Delta t=1$) as follows:

26 $$\begin{aligned}{} & {} u=e^{0.4696 \cdot \sqrt{1}} = 1.5993 \end{aligned}$$

27 $$\begin{aligned}{} & {} d=e^{-0.4696 \cdot \sqrt{1}} = 0.6252 \end{aligned}$$

**Fig. 2:**
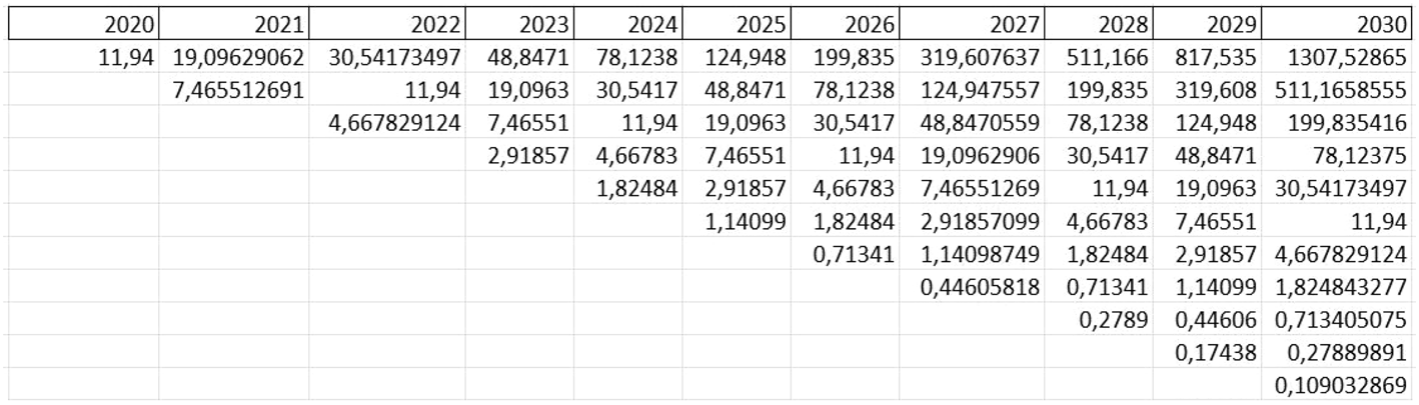
Projection of present value evolution in the future

**Fig. 3:**
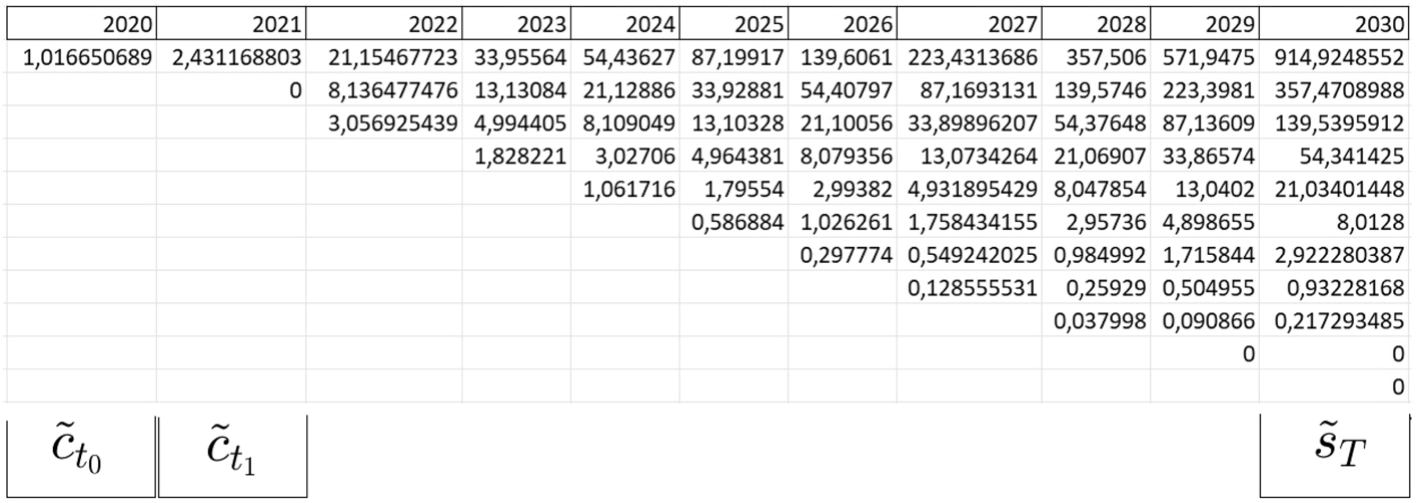
Calculation of $c_{t_0}$ following the binomial tree logic

To pursue the ROV we assume a risk-free rate ($r_f$) of 5.5%,^6^ the success probabilities of first and stages *p* and *q* both equal to 0.7, and $\rho (S_2,S_1)=0.73$. In fact, in the case of the broadband project, regarding the articles described above and the global score in Table 2, we calculate the $\gamma _{adj}$ as defined by Eq. 8. In this way, we obtain $\gamma _{adj} = 0.54$, whose root $\sqrt{\gamma _{adj}} = 0.73$. Firstly, we calculate the expected evolution of underlying assets (*PV*) from 2020 to 2030, as shown in Fig. 2. Then, we compute the binomial tree related to the option value, as shown in Fig. 3. Results show a project value $c_{t_{0}}={\EUR } \ 1.01665$ million, and, after multiplying it by success probability $p=0.7$, we subtract the planning investment to initiate the project ($I_0={\EUR } \ 0.5177$ million), and obtain a final RO value equal to € 0.19395 million.

All the parameters needed to pursue compound ROV are summarized in Table 6.

**Table 6:** Compound Real Option Calculation

Item	Description	Value
*PV*	Underlying asset	€ 11.94 million
$I_{0}$	Capital required for broadband plan	€ 0.5177 million
$I_{1}$	Capital required to proceed with building the network	€ 10.7250 million
$I_{2}$	Capital required to proceed with operating phase	€ 0.3452 million
$t_0$	Time instant for planning	0
$t_1$	Time instant for building	1 year
$t_2$	Time instant for operation	10 years
$\sigma$	Volatility of project returns	$46.96\%$
*u*	up movement	1.5993
*d*	down movement	0.6252
Period	years	2020–2030
$\Delta t$	Time variation	1 (yearly)
$r_{f}$	risk-free rate	$5.5\%$
$\pi$	risk-neutral probability related to up movement	0.4411
$1-\pi$	risk-neutral probability related to down movement	0.5588
*p*	Success probability of the construction phase	0.7
*q*	Success probability of the operation phase	0.7
$\sqrt{\gamma _{adj}}$	Revealed information parameter based on polarity score	0.73
$q^{pos}$	Revealed success probability transmitted from phase 1 to phase 2	0.919
$q^{neg}$	Revealed failure probability transmitted from phase 1 to phase 2	0.189
RO value €0.19395 million		

### Discussion of results: findings, implications, and limitations

Results show that the broadband projects evaluated through our methodology are financially attractive since the RO value is positive (equal to € 0.19395 million). On the one hand, thanks to the ROV, the proposed method can embed the riskiness in the project valuation. On the other hand, thanks to the use of AlBERTino, we can identify possible risks coming from the outside world (and expressed in natural language) and, consequently, evaluate whether or not a specific phase has been overcome. Our methodology allows to improve previous models and overcome some limitations concerning previous literature. For example, Burger-Helmchen (2009) determined the value of an investment in specific resources and competencies, taking into account the evolution of a specific industry (video games) when a new technology appears (a new console). But the data used were minimal. Such a model could have benefited from sentiment analysis, using AlBERTino, based on the data from many video games newspaper on new technology. Besides this specific example, the reasoning could be extended to several other organizational strategy questions following the survey of Driouchi and Bennet (2012) focusing on governance modes and management of multinational operations. However, this paper presents some limitations related to the case study. Specifically, we make some assumptions about the success probabilities, but a deeper analysis can be required to compute these parameters analytically. Furthermore, another limitation concerns using newspaper articles to improve the probability of success. In this toy model, articles from different sources have been used. However, it is clear that if this methodology took hold, it would be possible for some to polarize the published news. In this case, it could be a valid alternative to consider a part of newspaper articles and another part of indications/considerations of managers (always expressed in natural language); or in any case, subjects who have in-depth knowledge of the sector and could balance the polarization of external news.

Applying this methodology has implications for policymakers, given that funding must be gathered from public and private sources to implement broadband. Governments need to make public and private investors aware of the complexity embedded in these smart city projects so that they base their investment decisions on the outcome of assessment analyses that properly valuate these investments without underestimating them. This is a relevant point considering that public administrations often lack financial resources and need private support to pursue long-term investments associated, among others, with several of the 17 UN Sustainable Development Goals (SDGs). Specifically, UN Sustainable Development Goal 11 means to “make cities and human settlements inclusive, safe, resilient, and sustainable”. In particular, broadband investments are part of sustainable cities and human settlements since they can improve the performance of various technologies that make a city smart: smart traffic, safety, smart grid, and city intranet. The improvements happen through an optimization of the processes. People need a smart city to speed up their processes and their communication and live in a safe community. In this sense, broadband projects represent a good alternative to ride the digital era considering that two-thirds of the world population is expected to live in urban areas by 2050. This ongoing process started in 2008 when the global urban population exceeded the rural population for the first time in history. Moreover, the need for innovative broadband investments to increase the speed of navigation on the internet is emphasized by the Covid-19 pandemic since it has significantly increased the number of people working from home. In this sense, to maintain this drastic change in their habits and extend other smart city initiatives, broadband implementation becomes essential and an appropriate assessment of this type of smart city project. Moreover, throughout the result findings regarding the polarity scores on ROV, the organizations, such as the telecommunications industry, can benefit from our methodology to provide a reliable valuation tool for interested public or private investors and to encourage them to invest in telecommunications projects. This is because the results of the case study proposed in this work tend to appreciate the telecommunications projects highlighting their financial profitability for potential investors.

## Concluding remarks

The valuation of risky investments characterized by a high level of novelty and sequential nature is still an open issue in financial literature.

In most cases, the valuation of the uncertain projects is done through the compound Real Option Valuation (ROV), which can consider various types of uncertainty that can affect each investment stage. In particular, within the uncertain investments category, broadband project opportunities can be valued as real options to quantify the risks associated with the investment.

However, the compound ROV could be incomplete decision-making support to make an ex-ante valuation about the future project performance. If the investor should decide whether to invest in the future, s/he should also consider the market information (that can be extrapolated by newspapers or other sources) about the broadband market.

Considering their valuation difficulties, we propose a reliable methodology to price a typical broadband project that combines the ROV with sentiment analysis in this article. In this way, we can incorporate into the analysis part of what happens outside the company in the different investment phases and, on this basis, modify the probability of success. The information revealed was obtained by analyzing the news from some newspaper articles through AlBERTino, which produced a sentiment score that, through appropriate manipulations, was integrated into determining the probability of success.

We apply this methodology to a hypothetical case study based on likely data to show how to implement the model we proposed in this paper.

Moreover, our model could be helpful in public administrations because the valuation process followed stresses the managerial flexibility of broadband projects in an obvious way, something that can contribute to attracting more interest for innovative broadband projects, increasing their number and, consequently, raising the number of smart city initiatives, a target consistent with pursuing UN Sustainable Development Goal 11.

## Footnotes

1 As a source of detailed information on the broadband steps, we recommend the European Commission website: https://digital-strategy.ec.europa.eu/en/news/broadband-investment-guide.

2 See https://www.fhgov.com/Government/Current-Programs-Initiatives/Municipal-Broadband-Committee/BroadbandFeasibilityReport012520.aspx.

3 see page 128 of Feasibility Report for a Community Network.

4 The calculations to extrapolate the values of revenues and operating costs are shown in Sect. 3.1.

5 These results are obtained by converting in euro the results on page 6 of Feasibility Report for a Community Network.

6 The choice of $r_f=5.5\%$ has been made by considering the study of Charalampopoulos et al. (2011).
